# Reliability and validity of the Turkish version of the WHO-5, in adults and older adults for its use in primary care settings

**DOI:** 10.1017/S1463423619000343

**Published:** 2019-07-01

**Authors:** Erhan Eser, Celalettin Çevik, Hakan Baydur, Soner Güneş, Tayfun Alperen Esgin, Çağlar Söğüt Öztekin, Esen Eker, Ufuk Gümüşsoy, Gün Baris Eser, Beyhan Özyurt

**Affiliations:** 1 Department of Public Health, Manisa Celal Bayar University School of Medicine, Turkey; 2 Department of Nursing, Balıkesir University Faculty of Health Sciences, Turkey; 3 Faculty of Health Sciences, Department of Social Work, Manisa Celal Bayar University, Turkey; 4 Department of Public Health, Balıkesir University School of Medicine, Turkey; 5 Department of Public Health, Çanakkale 18 Mart University School of Medicine, Turkey; 6 Manisa Province Health Directorate, Turkey; 7 School of Social and Behavioral Sciences, Erasmus University, Netherlands

**Keywords:** Turkey, validity and reliability, WHO-5, Wellbeing index

## Abstract

**Background:**

This study aims to determine the psychometric properties of the World Health Organization Well-Being Index (WHO-5) Turkish version in Turkish adults and older adults.

**Methods:**

This is a multicenter cultural adaptation study carried out with 1752 participants. Internal consistency (by Cronbach’s alpha); Construct validity (by known groups and confirmatory factor analysis-CFI) and discriminant validity are evaluated stratified by adults and older adults. Cohen’s Effect Size is used in known groups and discriminant validity analyses.

**Results:**

Distribution properties of the WHO-5 Turkish version are in acceptable limits. Alpha values are 0.81 for adults and 0.86 for older adults. The variances of the 58.5% of the adults sample and 63.9% of the older adults sample are explained in Exploratory FA. Model fits (CFI) are satisfactory ( > 0.95) in both samples; but RMSEA is poor in the older adults sample (0.166) whereas it is acceptable (0.073) in the adults sample. Known groups validity and discriminant analyses are satisfactory in both adults and older adults.

**Conclusion:**

The WHO-5 Turkish version has a good measurement capacity, internal consistency and good model fits in both samples. The error values in the older adults group suggest that the results when testing older adults should be interpreted with caution.

## Introduction

According to the World Health Organization (WHO), the state of good health is described not only as the absence of any sickness or disability, but also as a complete sense of well-being in all psychological, bodily and social domains (Diener *et al*., 2009). Well-being describes the state of subjective well-being which consists of positive and negative aspects (Guðmundsdóttir *et al*., 2014; Barden *et al*., 2015).

The World Health Organization Well-Being Index (WHO-5), introduced by WHO in 1998, is one of the most widely used scales that broadly measure subjective well-being with a limited number of items (Warr *et al*., 1985; Bech *et al*., 1996; Topp *et al*., 2015). The WHO-5 is a generic scale which is used to evaluate the general mental well-being of persons (Hall *et al*., 2011; Bech, 2012) in clinical settings. The WHO-5 was reported to be one of the very frequently used scales that measure mental wellness and quality of life in Primary Care settings in different population groups such as adolescents and students (Yusoff *et al*., 2013; Christensen *et al*., 2015; Downs *et al*., 2017); pregnant women (Mortazavi *et al*., 2015); individuals using primary care services (Henkel *et al*., 2004; Saipanish *et al*., 2009; Guðmundsdóttir *et al*., 2014; Christensen *et al*., 2015); and in population-based studies (Khosravi *et al*., 2015). Two main studies clearly mentioned the use and the superiority of the WHO-5 for screening mental well-being in PHC settings: Henkel *et al*. (2003) observed that, being the briefest screening questionnaire (and therefore the most practical to use), the WHO-5 produced very high sensitivity (93%) and negative predictive values (98%) compared to the other questionnaires with standard cut-off points in their paper (Henkel *et al*., 2003), whereas Löwe *et al*. (2004) concluded that, all three questionnaires (The Hospital Anxiety and Depression Scale, the Patient Health Questionnaire and the WHO-5) performed well in screening of the depressive mood (Löwe *et al*., 2004). Following the presentation of the WHO-5 scale, the original version in English was translated into many other languages by the WHO Regional Office for Europe (Staehr, 1998; Topp *et al*., 2015) and by others (Awata *et al*., 2007a; Newnham *et al*., 2010; Krieger *et al*., 2014; Kong *et al*., 2016; Halliday *et al*., 2017; Bonnín *et al*., 2018). The clinical validity for WHO-5 was found to be more than sufficient (Awata *et al*., 2007b; Saipanish *et al*., 2009; Newnham *et al*., 2010; Hall *et al*., 2011; Guðmundsdóttir *et al*., 2014; Bech *et al*., 2018). Furthermore, it was found that the external (clinical) validity of the WHO-5 was not affected by existing comorbid psychiatric disorders, and that it was a substantial indicator of symptoms of depression (Mergl *et al*., 2007).

The WHO-5 index has been validated for different populations (Bech *et al*., 1996; Heun *et al*., 2001; Awata *et al*., 2007a; Saipanish *et al*., 2009; Lehmann *et al*., 2011; Bech, 2012; Lucas‐Carrasco, 2012; Yusoff *et al*., 2013; Guðmundsdóttir *et al*., 2014; Moon *et al*., 2014; Christensen *et al*., 2015; Khosravi *et al*., 2015; Uludag *et al*., 2016) but the psychometric properties of the previously translated (by the leading author of this paper) Turkish version were not investigated, leaving its validity and the reliability unknown.

The mean annual Family Physician Services use has increased from 1.1 to–2.8 per person since the early 2000s in Turkey (Akman, 2014; Republic of Turkey Ministry of Health General Directorate of Health Information Systems, 2017). This improvement of the primary care service use in Turkey also increases the necessity of rapid evaluation methods in primary care, focusing not only on physical-related disorders but also on mental well-being in both adults and older adults. Sound and psychometrically valid assessment tools like the WHO-5 would therefore satisfy the unmet need of evaluating mental well-being in primary care in Turkey.

This study aims to determine and explore the psychometric properties of the Turkish version of WHO-5 in both adults and older adults separately.

## Methods

### Subjects and data collection

Turkish-speaking subjects over 18 years of age, having intellectual competency for answering all items were recruited for the study (*n* = 1752). Being intellectually competent is defined as providing correct answers to the two simple questions about the age of the respondent, and the date of the interview. This is a multicentre study that covers the secondary analysis of the data from seven unpublished cross-sectional representative studies conducted with different hypotheses in two provinces (Manisa and Balıkesir) of Turkey. The sample sizes were calculated by using the patient data sets of the seven Family Health Centers in both provinces and the data were collected by interviewer-assisted questionnaires at the houses of the respondents in all of the seven cross-sectional studies in 2017. Three of the seven data sets belonged to adults and three belonged to older adults samples and one sample is a mixed sample of adults and older adults. Primary endpoints in three adult samples are low back pain, obesity and occupational health; and falls and PHC services accessibility for older adults samples. Adults and older adults mixed sample explores the hypertension prevalence. The demographic, morbidity and WHO-5 data from these seven studies were used in this study.

These studies are summarized in Table 1.

**Table 1.** The list of data sources used in this study

Data source / Study theme	Study sample and province	*n*	%
Low back pain	Adults’ sample, Manisa	238	13.6
Obesity	Adults’ sample, Manisa	300	17.1
Occupational health	Adult workers’ sample, Manisa	286	16.3
PHC services accessibility	Older adults’ sample, Manisa	167	9.5
Falls	Older adults’ sample, Manisa	275	15.7
Falls	Older adults’ sample, Balıkesir	300	17.1
Hypertension	Adults’–Older adults’ mixed sample, Balıkesir	186	10.6
Overall		1752	100.0

### Socio-demographic and Morbidity variables

Socio-demographic variables used in the studies were age, gender, level of education, marital status, social security status, and perceived financial status of the household and social class.

The other variables used in the statistical analysis were the ‘perceived change in health status compared to the previous year’ and ‘the presence of any chronic disease’.

#### WHO-5

The WHO-5 is a short and effective screening measure for detecting mental well-being. The WHO-5 index was translated into Turkish in 1999 by one of the authors of this study, printed in the Turkish official version of the scale, which is included in the website: ‘https://www.psykiatri-regionh.dk/who-5/who-5-questionnaires/Pages/default.aspx’ which refers to various language versions of the WHO-5 including Turkish version.

The translation/Turkish adaptation process followed the forward translations (two independent forward translations and generating consensus forwards version), back translation and the cognitive debriefing interviews on 10 lay subjects. The items of the WHO-5 are: I have felt cheerful and in good spirits (item 1); I have felt calm and relaxed (item 2); I have felt active and vigorous (item 3); I woke up feeling fresh and rested (item 4) and My daily life has been filled with things that interest me (item 5). The participants were asked to specify to what extent each of these five statements was true for them in the last 14 days. The items are scored between 0 (at no time) and 5 (all of the time). Therefore, the overall raw score varies between 0 (the absence of well-being) and 25 (the highest level of well-being). A 100-point scale score may also be calculated by multiplying the crude score by four. According to the scale instructions, Major Depression Inventory (ICD-10) should be applied to the patient if the scale score is less than 13. With this scale, individual change over time can also be monitored. A score change of 10% or more (increase or decrease) indicates a clinically meaningful change (Ware and Davies, 1995).

## Data analysis

### Psychometric analysis

Reliability and validity analyses of the Turkish version of the WHO-5 were conducted in this study. ‘Confirmatory approach’ was used in both reliability and validity analyses. This approach meant that the Turkish version would be tested against the one-dimensional original (index) scale structure. Analyses were run stratified by adults (18–64 years of age, *n* = 940) and older adults (65 and over, *n* = 812).

### Descriptive analysis

The descriptive results of the individual items and overall scores are presented by measures of mean and SD; and skewness, kurtosis and ceiling and floor effects. Maximum acceptable ceiling and floor effect was considered as 20% (Andresen, 2000).

### Reliability analysis

Reliability analyses were presented by ‘item analyses’ and ‘internal consistency’. In the item analyses correlation coefficients values (corrected for overlap) were obtained between each Item’s score and the total score, demonstrating the contribution of each of the items to the overall scale score. Internal consistency was tested with Cronbach’s alpha value. Cronbach’s alpha were deleted calculated for both the index scale and separately when each of the five items was deleted. If item deleted alpha values are expected to be smaller than the overall alpha value, this means that all five items contribute to the variance of the scale. Any ‘if item deleted’ alpha values greater than overall alpha value may refer to a problematic item. Alpha value 0.7 and over indicates a Satisfactory internal consistency (Nunnally and Bernstein, 1994).

### Validity analysis

Construct validity of the Turkish version of the WHO-5 was evaluated in the validity analyses. The construct validity was tested with known groups validity, discriminant validity, exploratory factor analysis (via Principal components analysis with Varimax rotation) and Confirmatory factor analysis (CFA). Comparative Fit Index (CFI) and Root Mean Square Error of Approximation (RMSEA) values were calculated in CFA. Acceptable fit values are 0.90 for CFI and 0.08 for RMSEA (Hooper *et al*., 2008; Kline, 2016). The known groups and discriminant validity of the instrument was tested with the mean difference between subgroups (Student’s t test), while the magnitude of the differences was presented with Cohen’s Effect Size (ES) statistic (Cohen, 1988b). ES value closer to 0.20 indicates a small effect, whereas 0.50 a medium and 0.8 and over a big effect (Cohen, 1988a). One-way ANOVA analysis was used in comparing three or more groups where parametric conditions are satisfied. Post-hoc comparisons were conducted using Tukey’s B test. The upper limit for type 1 error was taken to be 0.05 in the statistical analyses. The analyses were done by using ‘SPSS version 21.0 for Windows’ (SPSS Inc., Chicago, Il, USA) and Lisrel 8.54 (Joreskog & Sorbom, 2003).

### Ethical considerations

This study was approved by the Manisa Celal Bayar University Ethics Committee.

## Results

Seven different studies’ data were used in the analyses. 53.7% of the study group was between 18 and 64 years of age and the rest were aged 65 and over. This study presents the results of the psychometric analyses stratified for both adults (18–64 years of age) and older adults (over 65). The mean age of the respondents was 40.35 ± 12.43 (range: 18–64) for the adult group and 72.87 ± 6.43 (range: 65–97) for the older adults group. 14.7% of the adult group and 41.7% of the older adults group was male; illiteracy rates were 8.2% for adults and 25.0% for older adults, whereas the percentage of insufficient income was 31.9% and 17.2% for adults and older adults, respectively (Table 3). Overall raw WHO-5 Score was 13.78 ± 4.93 for the adults sample and 14.86 ± 5.17 for the older adults sample. Converted mean 0–100 scale scores were 55.14 ± 19.72 for the adult groups and 59.42 ± 20.70 for the older adults group. Major Depression Inventory (ICD-10) should be applied to 36.7% of the respondents in the adults sample and 30.8% in the older adults sample, since the raw WHO-5 scores were less than 13.

**Table 3.** Known groups and discriminant validity results

	Adults ( < 65 years of age, *n* = 940)				Older adults (65 and over, *n* = 812)			
Variables	*n*(%)	Mean difference	*P*(#)	cohen’s d	*n* (%)	Mean difference	*P*(#)	cohen’s d
Gender (male)	138 (14.7)	−1.6	0.387	0.06	339 (41.7)	7.7	0.000	0.39
Education (primary and over)	860 (91.8)	3.3	0.159	0.09	610 (75.0)	10.8	0.000	0.70
Marital status (single)	256 (27.3)	−1.4	0.343	0.06	352 (43.3)	−8.2	0.000	0.42
Income (inadequate)	299 (31.9)	−4.0	0.004	0.19	140 (17.2)	−9.6	0.000	0.68
Presence of any chronic illness (no illness)	376 (57.8)	5.5	0.000	0.31	147 (18.1)	6.8	0.000	0.26
Smoking (no)	458 (72.6)	0.2	0.922	0.01	671 (83.1)	−5.5	0.005	0.20
Alcohol intake (no)	345 (55.6)	−0.5	0.750	0.03	750 (93.1)	−10.5	0.000	0.26
Exercise (at least once a week)	189 (29.0)	3.1	0.065	0.14	424 (52.3)	13.1	0.000	0.69
Sleep problem (no problem)	236 (37.9)	6.6	0.000	0.33	333 (41.3)	6.8	0.000	0.33
Body Mass Index ( > 30.0)	180 (29.6)	−7.1	0.000	0.34	149 (23.2)	−4.5	0.030	0.29
Social relations (satisfied)	378 (64.2)	−0.8	0.601	0.04	651 (88.1)	13.6	0.000	0.44
Psychological status (good-satisfied)	492 (93.7)	12.6	0.001	0.29	151 (91.5)	10.7	0.024	0.36
Perceived health (medium-poor)	187 (22.8)	−3.2	0.021	0.17				

(Cohen, 1988b).

Cronbach’s alpha values were 0.83 for the overall sample; 0.81 for the adults sample and 0.86 for the older adults sample. In both samples, when item five was deleted, Cronbach’s alpha was higher than the general alpha value, but item total correlations (corrected overlap) were greater than 0.35 for all items in both adults and older adults samples, indicating that item five should not be considered a problematic item.

Exploratory Factor Analysis showed Keiser Meier Olkin (KMO) values to be 0.82 in both groups and cumulative exploratory variance percentage to be 58.5% in adults and 63.9% in older adults. This means that the sample size adequacy has been ensured for both groups, that the scale presents a one-dimensional structure and that the explained variance is above 50% (Table 2). Item loadings for each of the individual items were over 0.7 except for item five, which has lower item loadings in both adults (0.50) and older adults groups (0.69). CFA results indicated good fit indices for both the adults and the older adults samples: CFI values were over 0.95 in both samples. However, the RMSEA value was in acceptable limits (under 0.08) in the adults sample, whereas RMSEA was over acceptable limits (0.16) in the older adults sample. Error residuals of the items were in moderate levels and the commonalities with the overall scale in an adequate magnitude (Table 2).

**Table 2.** Results of WHO-5 item analyses, internal consistency, exploratory and confirmatory factor analyses in adults and older adults

	Adults ( < 65 years of age) (*n* = 940)				Older Adults (65 and over) (*n* = 812)		
WHO-5 items	Cor.(a)		C.Alpha(b)	FacL(c)	Cor.(a)	C.Alpha(b)	FacL (c)
1. I have felt cheerful and in good spirits	0.69		0.75	0.83	0.70	0.82	0.83
2. I have felt calm and relaxed	0.67		0.76	0.82	0.68	0.82	0.81
3. I have felt active and vigorous	0.72		0.74	0.85	0.73	0.81	0.85
4. I woke up feeling fresh and rested	0.61		0.78	0.77	0.70	0.82	0.81
5. My daily life has been filled with things that interest me	0.36		0.85	0.50	0.55	0.86	0.69
Overall Cronbach’s Alpha value	0.81				0.86		
**Exploratory Factor Analyses (EFA)**							
KMO		0.83			0.82		
Bartlett’s sphericity test		0.000			0.000		
Cumulative exploratory variance percentage		%58.5			%63.9		
**Confirmatory Factor Analyses (CFA)**							
Chi-square/DF		6.0			23.3		
RMSEA		0.073			0.166		
CFI		0.989			0.956		
NFI		0.986			0.954		
GFI		0.987			0.946		
Stand RMR		0.021			0.043		
**Item analyses of the CFA**							

(a) Item total correlations corrected for overlap; (b) If item deleted Alpha values; (c) Factor loadings generated by Principal Components Analyses; KMO: Kaiser-Meyer-Olkin Measure of Sampling Adequacy, RMSEA: Root Mean Square Error of Approximation, CFI: Comparative Fit Index, NFI: Normed Fit Index, GFI: Goodness of Fit Index, Stand.RMR: Standardized Root Mean Square Residual.

Table 3 presents known groups and discriminant validity findings for both age groups. In the adults group, those with insufficient income, with chronic diseases, sleep problems, having a Body Mass Index (BMI) of more than 30 and those with poor psychological status had a significantly lower scale score (*P* < 0.05). In the older adults group, the WHO-5 scores were worse in women compared to men; illiterate persons compared to educated ones; those having inadequate income compared to adequate income; those with chronic diseases compared to healthy persons; smokers compared to non-smokers; those with sleep problems compared to the people who do not have any sleep problems; those with high BMI values compared to normal weighted persons and those with poor psychological status (*P* < 0.05). Cohen’s effect size figures indicate that, in the older adults group the WHO-5 score was more sensitive to the known variables than in the adult groups (Table 3).

## Discussion

In this study, the psychometric properties of the Turkish version of the WHO-5 were found to be quite satisfactory. The study covered a wide range of psychometric evaluations except for sensitivity to change due to its cross-sectional design. There were two points that distinguish that distinguished our study from many other studies: Firstly, the sample pool consisted of individuals using primary health care services representing the community, and secondly, evaluations were done separately in adults and elderly individuals. The main reason for using stratified analyses was to take into account that self-assessment of mental well-being might differ between between adults and the elderly.

Illiteracy rates were 8.2% for adults and 25.0% for older adults in this study. Illiterate persons (especially those older adults living in the suburbs) are part of the community and should be included in the study for the sake of generalizability of the results to the Turkish population. So, the questionnaires were applied either via interviewer administration or in an interviewer-assisted way.

When we compared the WHO-5 score obtained from various studies and the WHO-5 scores obtained from our study, the average values obtained in various countries of Europe (De Wit *et al*., 2007; Klis *et al*., 2008; Gorter *et al*., 2010; Lehmann *et al*., 2011; Nicolucci *et al*., 2013; Bahrmann *et al*., 2014; Guðmundsdóttir *et al*., 2014) were between 62.5 and 66.4, which are higher than our results (WHO-5 mean values in the adults and the elderly group were 55.13 and 59.42, respectively, in our study). Similarly, in a German study (Bahrmann *et al*., 2014), the raw WHO-5 score was found to be 17.7, whereas the WHO-5 raw scores were 13.7 for adults and 14.9 in older adults in our study. These score differences between European countries may be explained mainly with the different socioeconomic conditions, which are worse in our study sample compared to the European samples. On the other hand, higher WHO-5 scores were obtained in other studies conducted on higher educated Turkish population compared to our study (Lehmann *et al*., 2011; Makine *et al*., 2011). Besides, our results are closer to the results of an Iranian study (Khosravi *et al*., 2015). These results also support our hypothesis of socio-demographic factors effecting mental well-being. By contrast, in a multicenter study (Nicolucci *et al*., 2013) which consists of 17 countries including Turkey, the poor mental well-being prevalence was found to be 19% if the cut-off point was set as 28 points in a 100-point scale, whereas this prevalence was 12.0% in adults and 10.8% in older adults based on the same cut-off point used in our study. This may be attributed to age, gender and socioeconomic differences between study samples.

### Study sample and distribution properties

KMO values obtained in the EFA for both the adult and older adult samples were greater than 0.50, confirming sample size adequacy. Ceiling and floor effects of the scale in both the adults and older adults groups were within acceptable limits (0.4%–2.2%), pointing to a remarkable measuring capacity of the WHO-5 in both samples. Skewness and Kurtosis values were more than sufficient, which indicates a normal distribution, eradicating any distribution- related uncertainty in the reliability and validity analyses (West *et al*., 1995). In both samples, when item five was deleted, Cronbach’s alpha increased over the general alpha value, but item total correlations (corrected overlap) were greater than 0.35 in both samples. This demonstrates that item five could not be considered a problematic item.

### Reliability and validity analysis

The alpha coefficients of both adults (0.81) and older adults (0.86) are over 0.70, pointing out a satisfactory internal consistency. These values are consistent with all literature findings (Awata *et al*., 2007a; De Wit *et al*., 2007; Saipanish *et al*., 2009; Makine *et al*., 2011; Ramona, 2012; Khosravi *et al*., 2015; Bonnín *et al*., 2018). If item deleted alpha values did not indicate any problematic items in older adults. In adults’ version, the only potential problematic item is item five, since the alpha value when item five is deleted is greater than overall alpha value (8.1 versus 8.6). Nonetheless, the item total correlation for item five (corrected overlap) was greater than 0.35, which indicates that item five is not a problematic item, and this eliminates any previous doubts. The three methods used to demonstrate the construct validity analyses were: ‘factor analyses’, the ‘known groups analysis’, and the ‘discriminant validity analysis’. Exploratory factor analyses of the Turkish version in both age groups have revealed a one-dimensional structure, as it was suggested in other language versions of the WHO-5 (Bech *et al*., 1996; Heun *et al*., 2001). Factor loadings obtained from exploratory factor analyses and the model’s variance explanation potential were found to be consistent with other studies in the literature (De Wit *et al*., 2007; Saipanish *et al*., 2009; Ramona, 2012; Khosravi *et al*., 2015). Due to some limitations of the exploratory factor analyses, CFA was also suggested in the literature (Guðmundsdóttir *et al*., 2014). The CFI generated by CFA was satisfactory ( > 0.95) in both adults’ and older adults’ samples, but the indices showing error residuals were found weaker in the older adults’ version compared to the adults’ version. RMSEA value of the adults’ version was 0.073, which is lower than the acceptable limit ( < 0.08) whereas the RMSEA and 0.166 in older adults. In other language validation studies, the fit indices were all in acceptable limits as ours, but the RMSEA was satisfactory only in the German diabetics study with 0.062 (De Wit *et al*., 2007). The RMSEA was, 0.104 in the Iranian version (Khosravi *et al*., 2015); 0.09 in men and 0.15 in women in the Icelandic version (Guðmundsdóttir *et al*., 2014); and 0.23 in the Australian English version (Halliday *et al*., 2017), which are all over acceptable limits. Looking at these results, we can confidently say that the Turkish version has an acceptable construct validity in adults. Some of the relations of known groups analyses agreed on in the literature have also been tested here as another way of showing the construct validity: The WHO-5 was found to discriminate between categories of age, gender, level of education, income and marital status. According to the effect sizes, the strongest discriminative variables were education and income.

### Discriminant validity

As the original structure of the scale showed an index (one-dimensional) feature, this study focused on clinical sensitivity findings in addition to item analyses. A similar approach was followed in the WHO-5 studies in other countries. In most of the studies in the literature, either ROC analyses were conducted through a parallel scale by assuming a cut-off point, or sensitivity and specificity values were calculated using suggested cut-off points for WHO-5, which are 28 and 50 points for 100-point scale; and 12–13 points for raw 25-point scale. Mainly two cut-off points (50 for 100-point scale and 12–13 for 25-point scale) are suggested in the literature (Henkel *et al*., 2004; Hajos *et al*., 2013; Firdaus, 2017; Halliday *et al*., 2017). Different cut-off values were also suggested such as 13 in an Australian study (Halliday *et al*., 2017) and 10 in a Europe and South Asia comparison study (Aujla *et al*., 2010) (Aujla *et al*., 2010) for raw scores (on 0–25-point scale); and 28 in 100-point scale (Lehmann *et al*., 2011).

## Strengths and limitations

The representative big cross-sectional sample obtained from diverse socioeconomic groups of the community is one of the main strengths of this study. The other one is the stratified analyses done for the adults and older adults that distinguishes our study from the many others.

The main restriction of our paper is the lack of ROC analyses. We did not apply ROC and sensitivity analyses since we could not use a reference test in this study. Instead, we applied discriminant analyses by dichotomizing the WHO-5 raw score by 13 (as suggested in the official instructions of the WHO-5). Existence of any non-communicable disease, exercise frequency, sleep problems, obesity, violence and abuse, the satisfaction with interfamily relations and perceived psychological and general health status were the variables used in these discriminant analyses. The Turkish version of the WHO-5 could discriminate all subcategories of these variables.

## Conclusion

To conclude, the distribution properties, measurement capacity and the internal consistency of the Turkish WHO-5 were sufficient and satisfactory in both the adults and older adults samples. Although the fit indices are acceptable in both samples in CFA, error residuals were out of acceptable limits in the older adults sample. So, we suggest that, the Turkish version of the WHO-5 can confidently be used in adults (18–64 years of age) whereas the results should be interpreted with caution for older adults.
